# Editorial: Planetary health challenges and physical activity

**DOI:** 10.3389/fpubh.2023.1240097

**Published:** 2023-07-13

**Authors:** Martin Schnitzer, Susan Houge Mackenzie, Martin Kopp

**Affiliations:** ^1^Department of Sport Science, University of Innsbruck, Innsbruck, Austria; ^2^Department of Tourism, University of Otago, Dunedin, New Zealand

**Keywords:** climate change, COVID-19, exercise, health economy, health tourism, physical activity promotion, sport

## Introduction

The positive effects of sport and physical activity (PA) on physical and mental wellbeing are well-documented [e.g., (1, 2)]. However, planetary health challenges, such as the recent COVID-19 pandemic and long-standing climate change issues, raise questions about how these crises effect sport and PA, both now and in the future. For example, how will climate change impact individuals' or specific population groups' engagement with sport and PA? Can sports and/or PA help counteract negative developments associated with planetary health challenges? These questions formed the starting point for launching this Research Topic focused on planetary health challenges, sport, and PA. Thus, the aim of this Research Topic is 3-fold: First, to identify which planetary health challenges influence sport and PA, and how. Secondly, to investigate strategies which have the potential to maintain or increase sport and PA levels in a changing environment. Thirdly, to examine economic perspectives on these questions, in order to identify how changing environmental conditions may influence potential changes in sport, PA, and health-related tourism behaviors.

## Aim 1

Schöttl et al. investigated how the COVID-19 pandemic, both during and after stay-at-home orders, influenced the PA behavior of people located in the Austrian, German, and Italian Alps. They found that, although participants reduced their PA during lockdowns, overall they resumed to their pre-pandemic sport levels relatively quickly as stay-at-home orders eased. However, participants who were more likely to have prolonged reductions in their PA included men, participants experiencing psychological distress, and individuals with decreased physical health or free time during the pandemic.

Litleskare and Calogiuri also examined PA-related behavior during the COVID-19 pandemic, specifically nature visits in Norway. Participants in this study reported using natural environments as an alternative to gyms and organized sports. As a result, an increase in nature visits during this crisis was reported, particularly among women, younger respondents, individuals from high-income households, and individuals exposed to long-term restrictions.

## Aim 2

Climate change is dramatically changing natural environments, especially in mountainous regions, which has the potential to impact how people engage in sport and PA in those affected environments. While there is evidence of reduced intentions to engage in winter sports in climate change affected environments, Niedermeier et al. found that, in the near-term, reductions in mountain sport activities due to climate change seems unlikely during summer seasons. However, further research is needed to assess if there may be changes in engagement with summer season mountain sports in the long term.

Other sports-related leisure providers also suffered from the pandemic, including sport organizers and other stakeholders of sports events. Kogler and Schöttl investigated changes in attendance at major sports events, media engagement with major sports, travel intentions, and the introduction of new sports offerings. During the COVID-19 pandemic, self-reported importance of attending major sports events decreased significantly in Alpine regions, and prevailing restrictions affected vacation planning for the majority of participants. Sports facilities and opportunities played an important role in the choice of vacation destination among the sample, and all participants reported trying new sports offerings during periods of pandemic restrictions.

## Aim 3

Regional sustainable development initiatives have attracted much attention in the wake of the COVID-19 pandemic. Wang et al. analyzed the impacts and mechanisms of government sports expenditure on regional sustainable development from the perspective of sports economic development. This study found that China's sustainable development presents clear ladder-like characteristics and the authors' highlighted regional imbalances and the inadequacy of coordinated regional green developments. Increasing public spending activated the consumption level of inhabitants. They concluded that government increases in public expenditure on sports can promote regional sustainable development, and that, with continuous improvement of regional economic development, the positive impacts of public sports expenditure continue to increase.

Not only sportspeople, but also sports providers are hit hard by planetary health challenges and crises due to curfews and/or the closure of (outdoor) sports facilities. For example, during the COVID-19 pandemic ski operators across Europe were forced to close down. Steiger et al. used a mixed method approach to determine how cable car operators in Austria dealt with these crises, and whether new experiences and established measures will lead to improved customer experiences in the future. Although, from an economic perspective, the winter season during the COVID-19 pandemic was disastrous, cable car operators reported positive experiences, such as gratitude from the local population, and that measures introduced led to further advancement of the technologically long stagnant industry. They concluded that sustainability has become a more important issue and the future will bring a stronger focus on qualitative development for this industry.

In addition the COVID-19 pandemic, climate change is fundamentally changing how active, nature-based leisure is facilitated. Valdivielso Martínez and Houge Mackenzie conducted semi-structured interviews with adventure guides and found that adventure guides had deeply meaningful connections to, and relationships with, the natural environments in which they worked. These adventure guides reported a heightened sense of environmental responsibility, and described how this often created “ethical dilemmas” in seeking to resolve tensions between their deep connections to nature and the unsustainable practices that their guiding work often entailed.

As much as our lives have been marked by planetary health challenges and global crises in recent years, and will continue to be impacted by these challenges in future, these studies suggest that the positive effects of sport and PA on body and mind, both individually and at collective levels, may provide a successful strategy to maintain or increase physical wellbeing and reduce psychological distress. Particularly during difficult periods, such as the COVID-19 pandemic, PA has proven to be an effective method for dealing with challenging situations, as well as instigating long-term innovations and adaptations that may benefit sport providers and participants in the long term. Planetary health challenges such as climate change will continue to strongly shape the fields of sports, PA, tourism and leisure in the years to come. Sustainable development of these industries will continue to drive innovative research and practice in future.

## Author contributions

All authors listed have made a substantial, direct, and intellectual contribution to the work and approved it for publication.

## References

[1] Daskalopoulou C Stubbs B Kralj C Koukounari A Prince M Prina AM. Physical activity and healthy aging: a systematic review and meta-analysis of longitudinal cohort studies. Ageing Res Rev. (2017) 38:6–17. 10.1016/j.arr.2017.06.003 28648951

[2] Werneck AO Stubbs B Kandola A Oyeyemi AL Schuch FB Hamer M. Prospective associations of leisure-time physical activity with psychological distress and well-being: a 12-year cohort study. Psychosomat Med. (2022) 84:116–22. 10.1097/PSY.0000000000001023 34611110

